# Exclusion of invasive predators triggers succession, competition and habitat diversification in a small mammal community

**DOI:** 10.1098/rspb.2025.0325

**Published:** 2025-07-09

**Authors:** K. E. Moseby, John Read, Katherine Tuft, Genevieve Hayes, Helen Crisp, Cat Lynch, Leanne Van der Weyde

**Affiliations:** ^1^ Centre for Ecosystem Science, University of New South Wales, Sydney, New South Wales, Australia; ^2^ The University of Adelaide - North Terrace Campus, Adelaide, South Australia, Australia; ^3^ Arid Recovery, Roxby Downs, Australia

**Keywords:** rodent, dasyurid, desert, Australia, *Notomys alexis*, *Pseudomys australis*, exclosure, safe haven, feral cat, fox

## Abstract

Invasive species can alter small mammal communities. We examined the abundance and demography of 10 Australian desert small mammals inside and outside a fenced reserve after the exclusion of invasive rabbits, cats and foxes. Over 26 years, we found evidence for a species succession response triggered by the removal of endogenous disturbance (predation), similar to exogenous disturbance caused by fire, mining and deglaciation. Smaller rodents responded within 2 years whereas larger rodents became more abundant within the reserve after 5 years, eventually outcompeting the smaller rodents. The dasyurid response was later and more muted. Captures of rodents inside the reserve reached up to 33 times higher than outside after high rainfall years, suggesting that invasive predators have a significant impact and suppress rainfall-induced population booms. Larger rodents expanded their realized niche into non-preferred habitats, and intraspecific competition and species diversity increased. Minimal differences in breeding, body mass or sex ratios between inside and outside the reserve suggested abundance increases were primarily due to release from predation pressure rather than increased resources. Succession was shaped by competition and differences in predator susceptibility influencing reinvasion timing. Results demonstrate that succession dynamics in small mammals can be triggered by the removal of endogenous disturbance as well as changes in vegetation structure.

## 1. Introduction

Desert small mammal communities are influenced by a range of factors including climate, resource availability, predation and competition [1–3]. Low rainfall creates competition for limited resources and drives divergence in traits such as body size, diet and spatial activity [4]. A characteristic of desert small mammal communities is dramatic abundance fluctuations in response to unpredictable periods of high rainfall and drought. Resource pulses trigger population booms in some species followed by crashes caused by resource limitation and/or predation [2]. Population fluctuations are also found in small mammals in mesic environments [5] but these are often more predictable due to strong seasonal variation, with cycles ranging from 3 to 5 years [6]. Globally, small mammal population fluctuations are strongly influenced by changes in individual survival and, to a lesser extent, rates of reproduction [7].

The natural processes structuring desert small mammal communities have been disrupted globally through habitat degradation, the introduction of invasive herbivores and predators, changes in fire regimes, clearance for agriculture and climate change [8]. These perturbations hinder the ability to discriminate between natural and human-induced changes. Exclusion experiments can assist with understanding natural processes such as interspecific competition and predation [9–12] but most exclosures are not landscape-scale (e.g. [13]) or only monitored over short periods [14]. Changes in community composition and abundance within exclosures are likely to be dynamic and occur over extended periods as seasonal conditions change, native species re-establish and respond at different rates, and the processes of natural competition and predation re-establish. These post-exclosure trajectories may be conceptually similar to the chronological species secondary succession events (*sensu* Clements [15] and Gleason [16] for plants) observed after exogenous disturbance and structural vegetation changes [17] from fire [18,19], farming [20], forestry, mining [21] and deglaciation [22].

Small mammal communities in the Australian deserts have been particularly affected since European colonization [23]. Desert mammals have one of the highest recent extinction rates, and the introduction of invasive predators such as the European fox (*Vulpes vulpes*) and feral cat (*Felis catus*) has been a major contributor [24]. These predators are a leading cause of translocation failure [25,26]. Rodents are highly susceptible to cat predation and both cats and foxes have their greatest impact on prey species within the critical weight range (CWR) of 35 g and 5.5 kg [23]. Evidence of these impacts is supported by predator exclosure and control programmes where populations of native CWR mammals have increased significantly after the exclusion or control of cats and foxes [12,25,27]. Invasive herbivores such as the European rabbit (*Oryctolagus cuniculus*) cause land degradation and elevate densities of invasive predators, facilitating hyperpredation. Domestic stock have further degraded desert ecosystems in Australia and even native macropods without management can become overabundant with resulting effects on wildlife [28]. These herbivores contribute to a reduction in food resources and habitat complexity, likely reducing breeding rates, suppressing boom periods and rendering small mammals more susceptible to predation.

The establishment of a large, fenced conservation reserve in arid Australia provided a unique opportunity to understand natural population dynamics and the impact of invasive species on an extant small mammal community. The Arid Recovery Reserve includes a 6000 ha fenced exclosure where introduced herbivores and predators (rabbits, domestic stock, cats, foxes) were removed and red kangaroo (*Osphranter rufus*) numbers reduced to negligible levels over 25 years ago. We investigated long-term trends and succession in 10 extant species of small (<60 g) mammals (rodents and dasyurids) using pitfall and Elliott trapping. *Dasyurids* are carnivorous marsupials that are characterized by their biting and cutting teeth and their relatively short lifespans. Common dasyurids in arid Australia include dunnarts (*Sminthopsis* spp.) and planigales (*Planigale* spp.) that feed mainly on invertebrates and generally weigh less than 20 g. We used Generalized Joint Attribute Models (GJAM) to assess dynamic interactions between species across areas inside and outside the reserve over 26 years, before and after the removal of rabbits, cats and foxes. We explored the influence of exclusion and climatic factors by comparing dynamic changes in small mammal abundance, diversity, body mass, breeding rates and sex ratios in two habitats. Changes in mass and breeding might be expected if resources increased inside the exclosure after the removal of key herbivores. Alternatively, if introduced predator exclusion was a significant catalyst for change then we expected primarily an increase in abundance. We predicted that small mammal population trajectories might follow a species succession response due to interactive effects such as reinvasion timing, variable susceptibility to introduced predators, intraspecific and interspecific competition, and habitat and environmental factors. Results provide an insight into the natural processes and interactions influencing desert small mammal communities prior to European colonization. Our study may assist with providing appropriate baselines for ecosystem restoration in arid areas and understanding human-induced impacts in other parts of the world.

## 2. Methods

### (a) Study site

**Figure 1.**
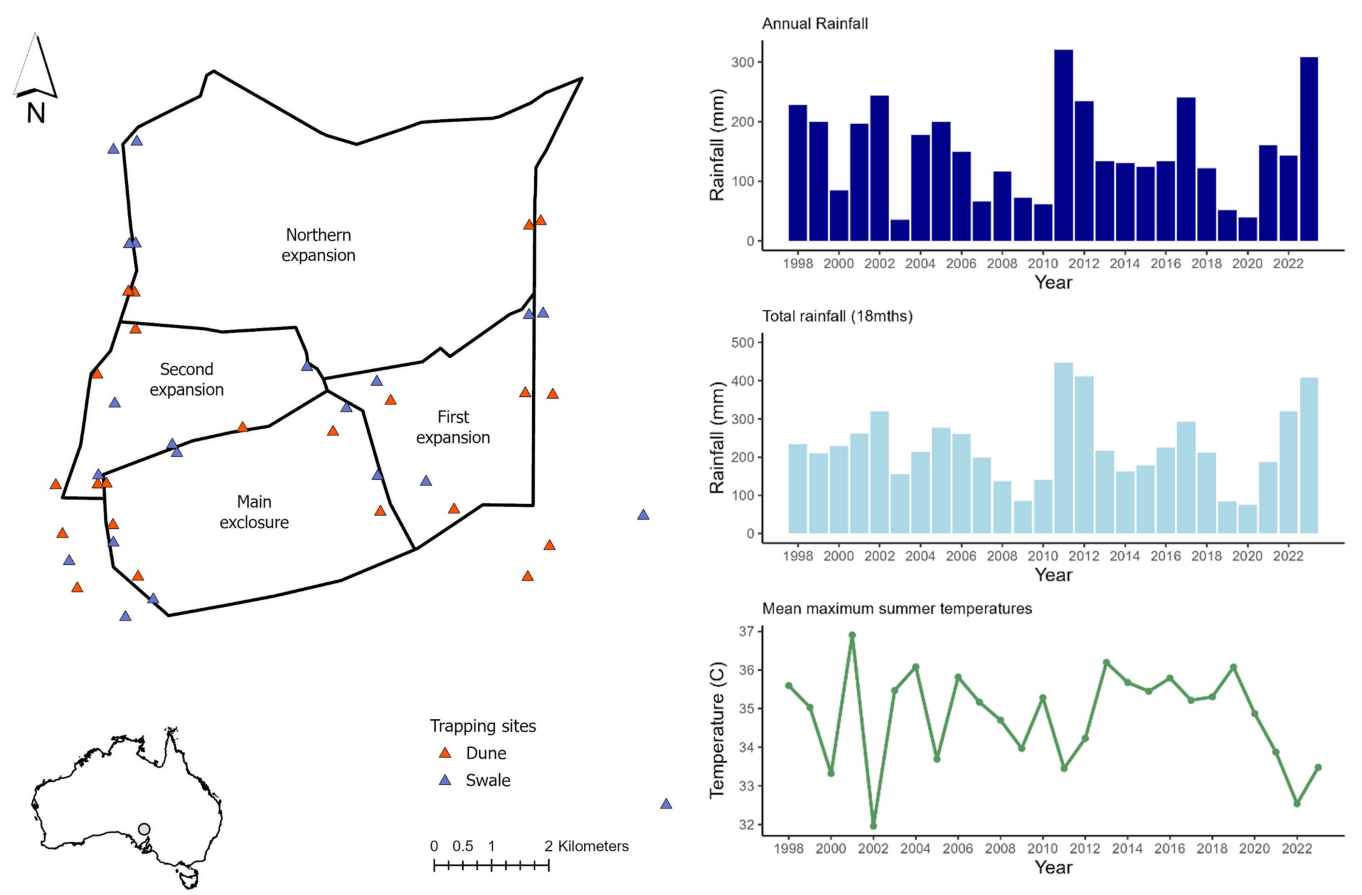
Location of trap sites inside and outside the Arid Recovery Reserve. Climatic conditions are shown as annual rainfall at the reserve, accumulated rainfall over 18 months prior to mid-March (average survey date) and the mean maximum daily summer temperatures from November to February prior to each survey.

The study was undertaken at the Arid Recovery Reserve in northern South Australia between 1997 and 2023. Arid Recovery is situated on Kokatha land and comprises six fenced paddocks (12 300 ha), of which four (6000 ha) are kept free of invasive rabbits, foxes and feral cats through a 1.8 m high netting exclusion fence ([29]; figure 1). Native kangaroo abundance is managed below 1 per km^2^. Rabbits, cats and foxes were removed from the Main Exclosure in late 1998, the First and Second Expansions in 2000, and the Northern Expansion by 2001.

The Arid Recovery habitat is dominated by sand dunes supporting mixed *Acacia* shrublands, and clay interdunal swales supporting chenopod shrubs such as low bluebush (*Maireana astrotricha*) and bladder saltbush (*Atriplex vesicaria*). Rainfall is unpredictable with a long-term average of 146 mm per annum [30]. Regional studies have recorded 55 species of extant reptiles and 10 species of native small mammals [31]. Locally extinct mammals were reintroduced to the reserve in 1999 (greater stick-nest rat, *Leporillus conditor*), 2000 (greater bilby, *Macrotis lagotis*), 2001 (burrowing bettong, *Bettongia lesueur* and Shark Bay bandicoot, *Perameles bougainville*), 2018 (western quoll, *Dasyurus geoffroii*) and 2022 (kowari, *Dasyuroides byrnei*).

### (b) Trapping

Small mammal trapping was undertaken between 1998 and 2023 with 43 different sites trapped at least once over the study (sites × session *n* = 535, figure 1, electronic supplementary material, figure S1). Sites inside and outside the reserve were trapped annually but the sites, configuration and method changed slightly over time. The mean number of sites trapped per year was 21.3 (19–39) and all inside and outside sites in any sampling year were trapped simultaneously. Sites were established in dune and swale habitat with inside and outside sites usually paired in the same swale or dune. Both habitats were trapped annually until 2006 after which they were alternated. Captured mammals were identified, weighed to the nearest 0.1 g, sexed, marked with a permanent marker on the tail, and released.

### (c) Capture rates

We investigated the influence of treatment (inside/outside) and habitat type (dune/swale) on unique captures over time for seven species with sufficient captures (table 1). Additionally, for each species, we divided the mean counts per year at inside sites in their preferred habitat by outside sites to assess the magnitude of difference. In years where we had no captures of a species at outside sites, we set the magnitude at 1 to provide a conservative no difference.

**Table 1.** Total individuals captured for each mammal species using pitfall and Elliott traps between 1998 and 2023 inclusive. Preferred habitat and mean body mass (g) of all captured individuals (adults and subadults) are shown. * Invasive, R = Rodent, D = Dasyurid.

species	taxon	Latin name	total captures	treatment		preferred habitat	body mass (g)
				inside	outside		
plains mouse	*R*	*Pseudomys australis*	855	769	86	swale	35.7
spinifex hopping mouse	*R*	*Notomys alexis*	4577	4145	432	dune	32.2
desert mouse	*R*	*Pseudomys desertor*	10	6	4	swale	21.8
forrest’s mouse	*R*	*Leggadina forresti*	69	40	29	swale	16.5
house mouse*	*R*	*Mus musculus*	1784	1020	764	swale	11.9
Bolam’s mouse	*R*	*Pseudomys bolami*	1047	908	139	both	11.7
stripe-faced dunnart	*D*	*Sminthopsis macroura*	184	142	42	swale	11.1
sandy inland mouse	*R*	*Pseudomys hermannsburgensis*	18	12	6	swale	10.6
fat-tailed dunnart	*D*	*Sminthopsis crassicaudata*	163	76	87	swale	9.3
Giles’ planigale	*D*	*Planigale gilesi*	5	3	2	swale	5.2

### (d) Species–environment interactions

To assess the influence of environmental factors (density independent) and competition (density dependent) on the relative abundance of the seven species between inside versus outside sites, we used GJAM. We also assessed how environmental factors influenced predicted abundance at carrying capacity (steady-state abundance) [32]. Modelling was conducted using the GJAM package and supplemental functions in R [33] using the gjamTime function to model dynamic data. We modelled relative abundance using individual captures and trap effort per combination of treatment (inside versus outside) and habitat (dune and swale), leading to four independent models. We only included species captured in more than 6 years of trapping.

Climatic conditions such as rainfall and temperature affect available resources and survival of desert mammals, influencing abundance and population growth [2,4,34]. We used accumulated 18 month rainfall and mean maximum daily temperatures over the four hottest months as climate predictors. Spatial differences in abundance between sites may be influenced by invasive or native species density, rainfall patchiness, vegetation or human disturbance. As we were limited in available data to represent these factors, we used latitude and longitude as proxies to represent spatial variability. We chose our predictors *a priori* to provide inference on these key environmental predictors affecting species–environmental interactions (details in electronic supplementary material, figure S2). All environmental predictors were standardized to have a mean of 0 and standard deviation of 1. As our data were counts of individuals, we used the discrete abundance (DA) specification allowing for species interactions to be specified. We set all rodent species to potentially compete with each other in all habitats, and the same with dasyurid species, by setting alpha parameter priors (−1, 0). However, we set rodents and dasyurids to have no interactions, based on their differing ecology and diet. The two dasyurids in this study primarily have an invertebrate diet [35], whereas the rodents are primarily herbivorous and granivorous [36,37].

Rodents can rapidly increase in abundance but our time steps were 1 year, so we set wide priors for density-independent environmental parameters (rho) enabling large changes in abundance to be modelled. We allowed a maximum of 300% change in population growth rates (−3, 3) per time step for both rho intercepts and predictor coefficients (change per 1 s.d. change in a driver) due to potential rapid changes in abundance. We used 30 000 iterations (including a burn-in of 20 000) for all models. Model convergence was checked by visually assessing parameter chains and diagnostic plots of posterior parameter estimates, and model fit was checked by assessing root mean square prediction error for all individual species and plots of observed and predicted estimates. Significant effects of predictors on growth rate (rho) were assessed using 95% Bayesian credible intervals that did not overlap zero. We estimated carrying capacity (equilibrium or steady-state abundance) and potential nonlinear relationships between species and environmental gradients (electronic supplementary material, figure S3).

Finally, we compared changes in competitive (density-dependent) interactions for each species within each habitat following the exclusion of introduced predators and herbivores. We followed Collins *et al*. [38] by assessing changes in competition (both intraspecific and interspecific) using mean alpha values between each species, as well as estimating a net change across summed changes in interspecific competition. We compared the modelled competitive interactions between individual species inside versus outside the reserve, after accounting for environmental effects. Inside sites were subtracted from paired outside sites, indicating changes in alpha parameter estimates (density-dependent mechanisms).

### (e) Diversity

Species diversity was measured using Hill numbers [39] using species richness and species relative abundances for each of our four treatment groups (inside dune (ID), outside dune (OD), inside swale (IS) and outside swale (OS)). We calculated three Hill numbers, species richness (*q* = 0), Shannon diversity (*q* = 1) and Simpson diversity (*q* = 2), using the package iNEXT [40]. We used species incidence frequencies to allow for comparisons across sampling effort as this varied between all groups, using rarefaction and extrapolation curves of summed sampled sites across all years. Sampling effort reflects the number of sites sampled pooled across all years for each group (ID 157, IS = 157, OD = 117, OS = 103).

### (f) Body mass

We modelled changes in body mass in response to each of our four treatment groups for three rodent species where sufficient sample size existed: spinifex hopping mouse (*Notomys alexis*), Bolam’s mouse (*Pseudomys bolami*) and plains mouse (*Pseudomys australis*). We evaluated the effect of climate (rainfall and temperature described above) and change over time as predictors of body mass, and included these as interaction effects with the treatment groups. We used linear mixed models (LMM) with our dependent variable body mass (log-transformed as necessary) fitted with a Gaussian error structure. Trap site was included as a random effect. Body mass analysis was restricted to adult males (hopping mouse ≥ 27 g [10]; Bolam’s mouse ≥ 9 g [34]; plains mouse ≥ 30 g [41]), to exclude pregnant females and juveniles. Only mass for the first capture of an individual within a survey was used. We used the glmmTMB package [42] using restricted maximum likelihood, and used plots of residuals to evaluate model fit using the DHARMa package [43].

### (g) Breeding rates and sex ratios

We modelled breeding rates of hopping mice and Bolam’s mice (proportion of juveniles captured per site) and sex ratios (proportion of male captures). We followed the same time and climate covariates as interaction effects with treatment groups described above. As our response variable was a proportion generated from relatively small sample sizes, often with a high number of zeros, we used the ordbetareg package [44] with a minimum of 4000 iterations and 2000 iterations as a warm-up for each model. Posterior distributions were checked to evaluate model fit. All models were conducted in R [45]. Estimated marginal means and contrasts (using Tukey adjustment of *p*-values) were conducted using emmeans [46].

## 3. Results

### (a) Capture data

A total of 226 512 (94 680 pit traps and 131 832 Elliott traps) trap nights were conducted over the 26 years between 1998 and 2023 inclusive. Seven rodents and three small dasyurid species (excluding reintroduced mammals) were recorded (table 1), with 8712 individuals from 9741 captures. Captures of desert mouse (*Pseudomys desertor*), sandy inland mouse (*Pseudomys hermannsburgensis*) and Giles’ planigale (*Planigale gilesi*) were insufficient for analysis. Body mass distributions are in electronic supplementary material, figure S1.

### (b) Abundance over time

**Figure 2.**
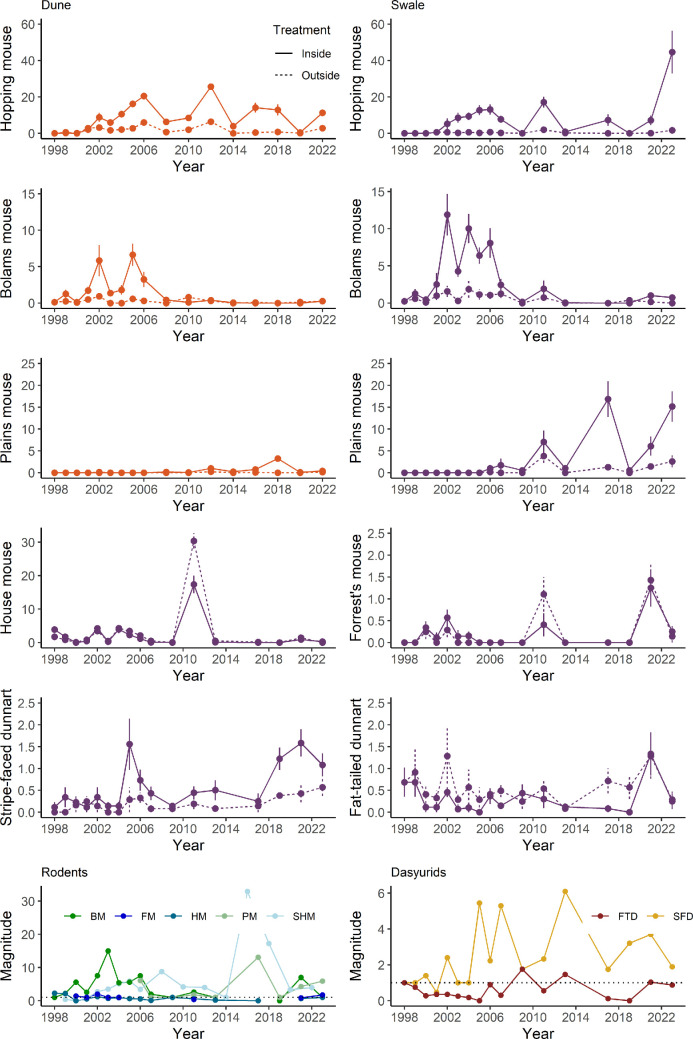
Change in raw mean captures (±1 s.e.) of seven small mammals at Arid Recovery from 1998 to 2023 at inside and outside sites from dune (orange) and swale (purple) habitats. Capture rate is standardized per 48 trap nights (standard number of trap nights since 2016). Captures for house mouse, Forrest mouse and the two dasyurids were too low at dune sites and were not included; *y*-axis scale differs considerably between species in order to show inside/outside effects. Magnitude graphs for rodents and dasyurids show differences in mean capture rates at inside sites relative to outside sites for each species’ preferred habitat.

No differences were observed in small mammal captures inside versus outside the reserve at the start of the study (figure 2). Over the next 26 years, four rodents and one dasyurid exhibited increases in abundance inside versus outside the reserve, one rodent and one dasyurid exhibited no change. The timing, magnitude and persistence varied between species.

The first species to respond were the smaller introduced house mouse (*Mus musculus*) and Bolam’s mouse, with capture rates increasing at inside sites relative to outside sites after only 1−2 years (figure 2; [10]). House mouse captures more than doubled in the first 2 years inside the exclosure but then remained similar to outside sites, except for 2011 when the species experienced a rainfall-induced boom which was significantly higher at outside sites. Captures of Bolam’s mouse were up to 15 times higher at IS sites and 5 times higher at ID sites between 2 and 9 years after reserve establishment, but then declined significantly after 2006 and were at similar abundance inside and outside. The decline in Bolam’s mouse captures corresponded with an increase in the larger rodent species, spinifex hopping mouse and plains mouse. Hopping mice and plains mice were not captured until 2 and 7 years after reserve establishment, respectively, but both species then increased rapidly at inside sites and remained higher for the rest of the study. At peak times, an average of 20 individuals were recorded at each inside dune (hopping mouse) and swale (plains mouse) site. The Forrest mouse (*Leggadina forresti*) was observed almost exclusively in swale sites and captures remained low, with no difference between inside and outside sites. The fat-tailed dunnart (*Sminthopsis crassicaudata*) fluctuated at consistently low rates with higher captures at OS sites in 2017 and 2019 during dry years. The stripe-faced dunnart (*Sminthopsis macroura*) generally exhibited low captures (<2 per site) but gradually increased at inside sites with more than a fourfold difference after 8 years. There were very few captures of house mouse, Forrest mouse and dasyurids in dune sites (non-preferred habitat; table 1), limiting any analysis.

Once species were established, native rodents had a higher magnitude difference between inside and outside sites than either dasyurids or the invasive house mouse, and larger rodents had a larger magnitude difference than smaller ones (figure 2). Hopping mice exhibited the greatest and most consistent magnitude difference (4–33 times more average captures at inside sites) and plains mice had a consistent magnitude response of 4–13 times. These differences were most pronounced after wet years. Bolam’s mice had a peak magnitude of difference of 15 times and stripe-faced dunnart 6 times.

Both larger rodent species expanded into non-preferred habitats. Hopping mice prefer dunes but captures were also higher at inside swale sites in most years after population establishment. Similarly, plains mice commonly inhabit cracking clay swales [47], but inside the reserve they were regularly trapped at dune sites once they had been established for more than 5 years (figure 2).

### (c) Growth rate (density-independent) effects with environment

**Figure 3.**
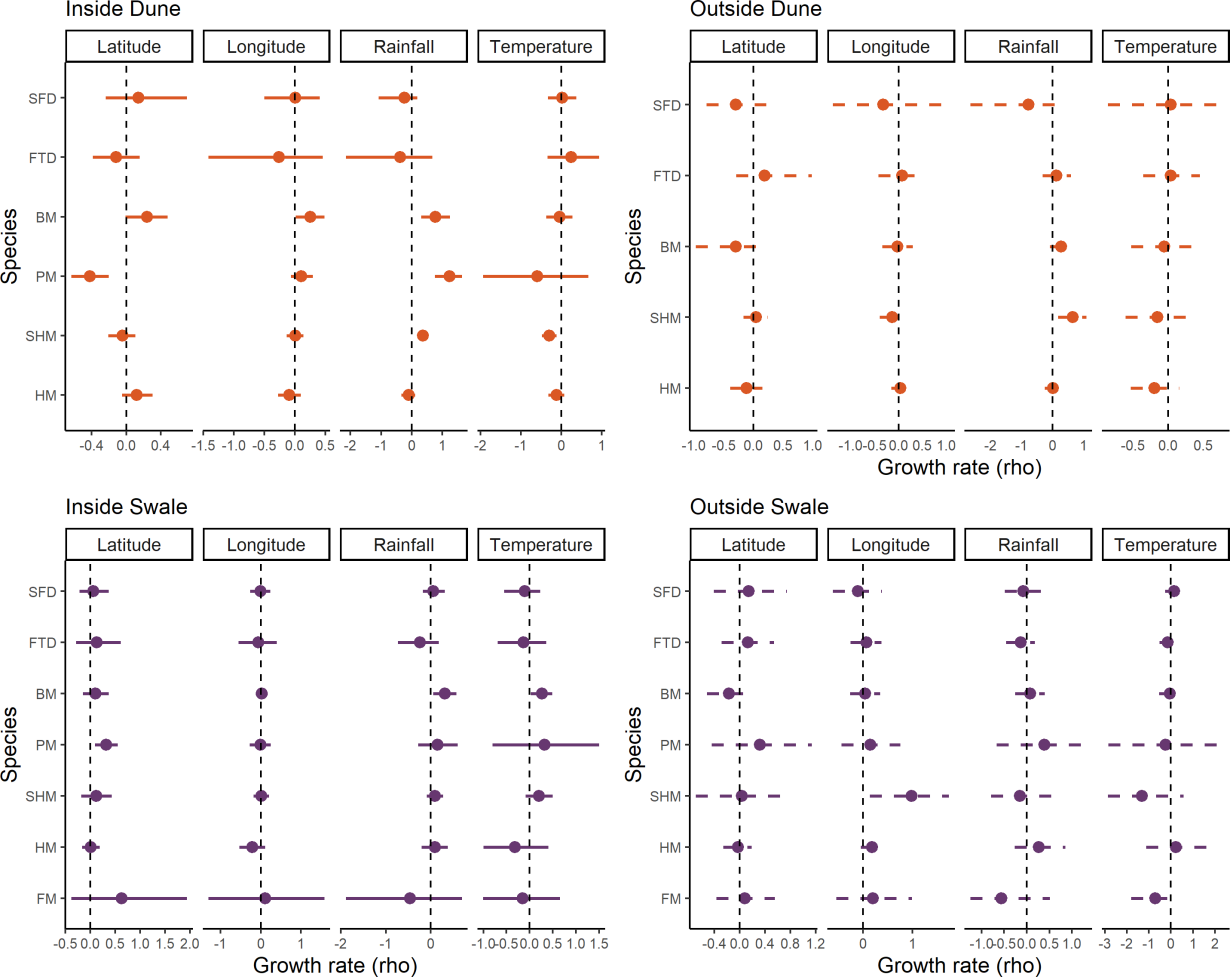
Density-independent effects of the environment on mean species growth rates (95% Bayesian credible intervals) in each of the four treatment groups. Stripe-faced dunnart (SFD), fat-tailed dunnart (FTD), Bolam’s mouse (BM), plains mouse (PM), spinifex hopping mouse (SHM), house mouse (HM) and Forrest’s mouse (FM). Inside sites are solid lines and outside sites are dashed lines.

At ID sites where hopping mice had the strongest increase in abundance (figure 2), growth rates were positively influenced by 18 month accumulated rainfall and negatively influenced by high summer temperatures (figure 3). Similarly, the same response to rainfall was observed for the Bolam’s mouse and plains mouse at ID sites (figure 3). Abundance of the Bolam’s mouse was greatest at IS sites, and rainfall and summer temperatures were found to be significant positive drivers of growth rates. Abundance for all species was generally low at outside sites and no climatic drivers were found to affect growth rates for any species, except hopping mice, where growth rates were higher following high rainfall periods in the dunes. Growth rates for some species differed spatially at inside sites (figure 3).

### (d) Species competitive interactions (density dependent)

**Figure 4.**
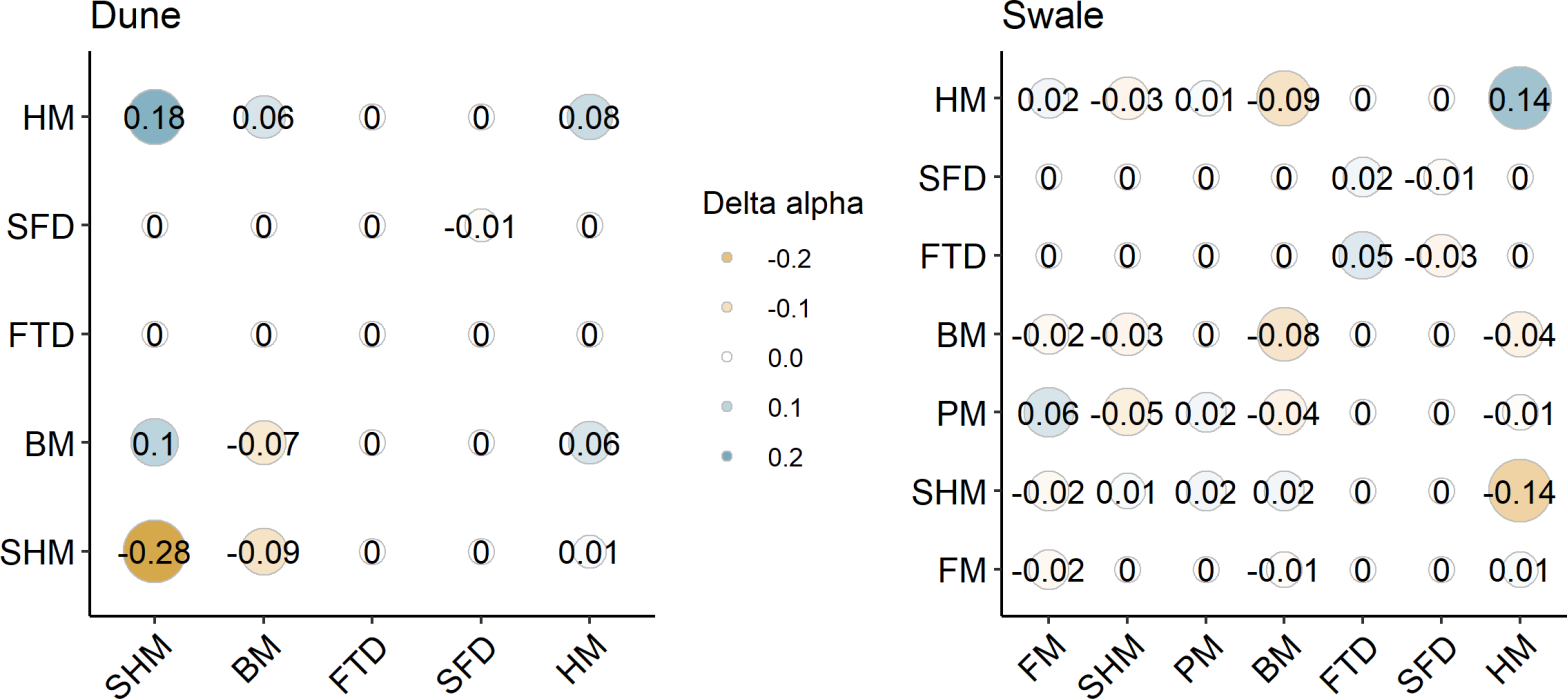
Species to species effects (*y*-axis on *x*-axis) when comparing outside to inside. Alpha coefficients are for species pairs where outside sites are subtracted from inside sites: blue indicates decreased competition and gold indicates increased competition at inside sites. Stripe-faced dunnart (SFD), fat-tailed dunnart (FTD), Bolam’s mouse (BM), plains mouse (PM), spinifex hopping mouse (SHM), house mouse (HM) and Forrest’s mouse (FM).

After accounting for environmental effects, hopping mice exerted the greatest competitive effects on themselves and other rodents (figure 4; electronic supplementary material, figure S3). Intraspecific competition in hopping mice was higher at inside sites while intraspecific competition in house mice was lower at inside sites. When considering interspecific competition, at ID sites where hopping mice were in high abundance, Bolam’s mice experienced higher competition from hopping mice but lower competition from house mice (figure 4; electronic supplementary material, figure S3). At IS sites, Bolam’s mice experienced higher competition from house mice, which in turn experienced higher competition from hopping mice, which experienced more competition from swale-preferring plains mice.

### (e) Net competitive interactions between species

**Figure 5.**
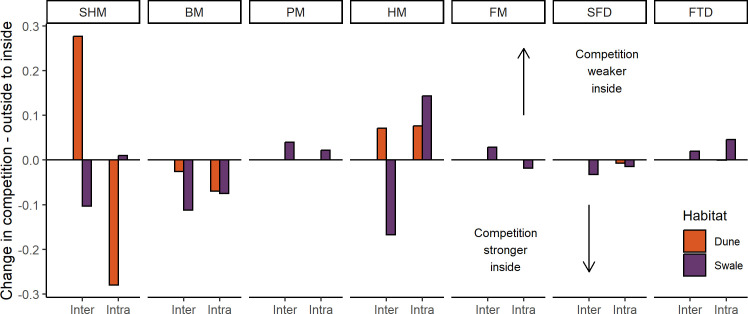
Interspecific and intraspecific competition changes moving from outside to inside sites in dune and swale habitats for five rodents and two dasyurid species. Negative changes imply competitive interactions increased and positive changes show that competitive interactions decreased. Changes for plains mouse (PM) and Forrest’s mouse (FM) were not calculated for dune sites. Spinifex hopping mouse (SHM), Bolam’s mouse (BM), house mouse (HM), stripe-faced dunnart (SFD) and fat-tailed dunnart (FTD).

Overall, competitive interactions between species increased within the reserve for nearly all species, except for the two largest species, hopping mouse and plains mouse, in their preferred habitat (figure 5; electronic supplementary material, figure S3). At inside sites, larger species were more influenced by intraspecific competition and smaller species by interspecific competition. For the larger hopping mice, the rapid increase in abundance and low competition from other species meant intraspecific competition (or resource limitation) was the main limiting factor in their preferred habitat (figure 5). For the smaller Bolam’s mouse at IS sites, interspecific competition appeared to be a major factor affecting abundance but intraspecific competition increased until 2005. Interspecific competition was also a larger factor affecting the abundance of smaller house mice once inside compared to the small but less so for the small Forrest’s mice. Competition was of lower importance for the two dasyurid species.

### (f) Carrying capacity

Hopping mice, Bolam’s mice and plains mice population growth had a significant positive relationship with rainfall (electronic supplementary material, figure S2), yet at predicted equilibrium, nonlinear distributions with rainfall were apparent for hopping mice and Bolam’s mice (electronic supplementary material, figure S1). These nonlinear effects were most evident at ID sites, where high abundance and competitive interactions may limit carrying capacity. A strong nonlinear effect of predicted equilibrium was observed at OD sites for the hopping mouse. Abundance still had a strong relationship with rainfall (electronic supplementary material, figure S2) but population abundance and competitive effects were lower, suggesting other factors outside the exclosure (e.g. predation) may limit growth in wet years.

### (g) Diversity

**Figure 6.**
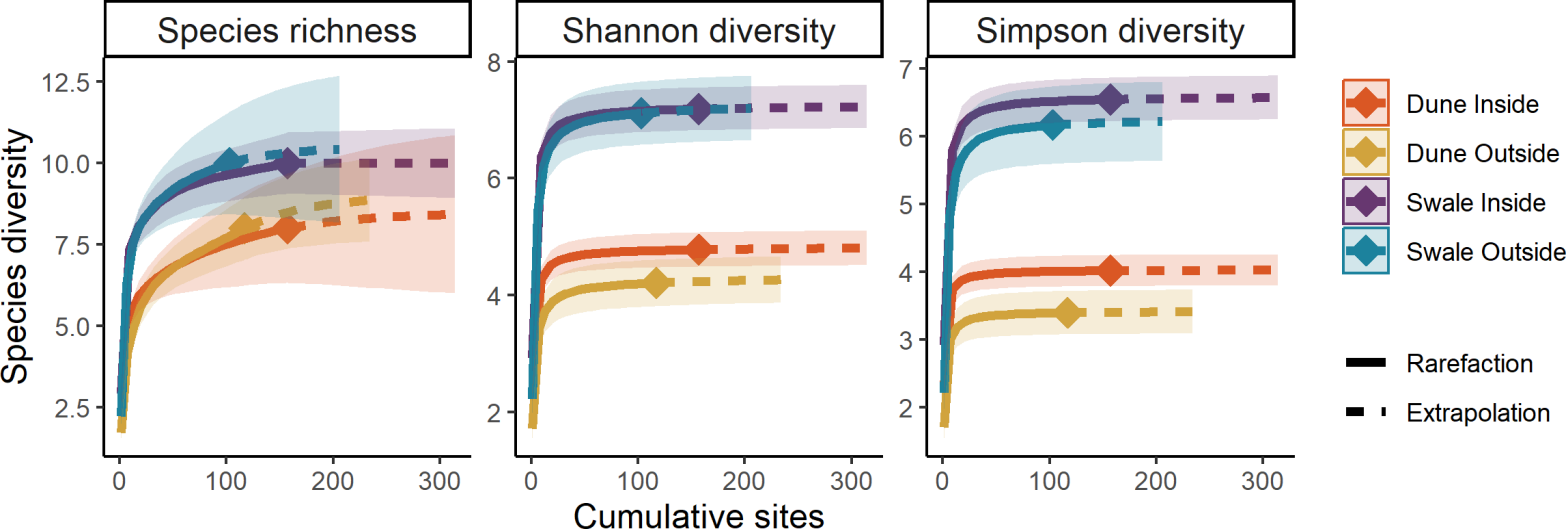
Species diversity metrics as calculated by Hill numbers for each treatment and habitat group for all small mammals trapped from 1998 to 2023. Diversity metrics are plotted along cumulative sampling effort showing rarefaction and extrapolation curves. Shaded regions show 95% bootstrapped confidence intervals.

ID sites had significantly higher Simpson and Shannon diversity than outside. The Simpson diversity index exhibited the highest divergence between inside and outside sites and reflects higher abundance of dominant species (figure 6). Species richness was similar inside and outside highlighting that most species were detected over the 26 years of sampling (figure 6).

### (h) Body mass

Rainfall and habitat had a stronger effect on body mass than temperature or treatment for hopping, plains and Bolam’s mice. A total of 1725 individual adult male hopping mice were captured, with the majority captured at ID sites (*n* = 851). The heaviest males were caught at ID sites (33.3 ± 0.3 g) and the lightest at both IS (32.8 ± 0.3 g) and OD sites (32.1 ± 0.5 g) when at mean rainfall, temperature and time periods, but no significant difference was found. Although significant interactions between treatment groups and rainfall were found (*Χ*^2^ = 42.38, d.f. = 3, *p* < 0.01), there were no significant differences between inside and outside sites within the same habitat. Body mass increased in response to higher rainfall in all dune groups, did not change in IS sites and declined in OS sites (electronic supplementary material, figure S4). A significant interaction between time and treatment groups was found (*Χ*^2^ = 15.62, d.f. = 3, *p* < 0.01); however, this difference was only between habitats for the inside sites, with body mass showing an increase over time at ID compared with a declining trend in body mass at IS sites.

Adult male Bolam’s mouse weighed on average 11.7 g (s.d. = 1.4; range 9–16.5). A total of 518 individuals were modelled, with the majority found inside (dune = 122; swale = 315). As with hopping mouse, body mass varied in response to increasing rainfall but the response varied between groups (*Χ*^2^ = 12.32, d.f. = 3, *p* < 0.01). Body mass at swale sites and ID sites generally did not change in response to rainfall. However, there was a strong positive association with increasing rainfall at outside sites (electronic supplementary material, figure S4).

A total of 369 individual adult male plains mice were caught, with a mean weight of 39.5 g (s.d. = 6.1, range 30–59.6). Males were found primarily inside (dune = 45; swale = 285) so the outside dune group was dropped from the analysis. At mean rainfall, temperature and time periods, plains mouse weighed slightly less at the ID sites (38.1 ± 2) compared with either of the swale sites (inside 39.5 ± 0.7 g; outside 40.7 ± 1 g).

### (i) Breeding rates

The proportion of juvenile to adult captures varied in response to temperature and time for hopping mice but no trends were detected in Bolam’s mice. The proportion of juvenile hopping mice caught in each treatment group (sampled sites: ID = 136, IS = 93, OD = 76, OS = 25) was highly variable over the sampling period, ranging from 0 to 100% at some sites, but were on average less than 30% when at mean climate predictors and time periods. The proportion of juveniles caught declined with high summer temperatures except for OS sites but this is likely related to fewer captures (electronic supplementary material, figure S4). The proportion of juvenile captures increased over time at all sites except OS and trends were significantly stronger at inside sites. For Bolam’s mouse (sampled sites: ID = 62, IS = 27, OD = 79, OS = 35), the percentage of juveniles were very low (<10%). There were no significant interactions between treatments and any predictors (electronic supplementary material, figure S4).

### (j) Sex ratios

The proportion of male captures did not vary in response to rainfall, temperature or time for either hopping or Bolam’s mouse (electronic supplementary material, figure S4). The proportion of male hopping mice at swale sites was on average higher (inside = 60%; outside = 62%) than dune sites (inside = 47%; outside = 49%) when at mean predictors. However, there were no significant interactions between male captures over time or in response to increasing rainfall or higher temperatures between habitats or inside and outside sites. Similarly, there were no main or interaction effects in the proportion of male Bolam’s mice between the treatment groups (ID = 62, IS = 79, OD = 27, OS = 35) and time or climate predictors, and trends were similar in both habitats and inside and outside sites.

## 4. Discussion

We found evidence for a succession response in a desert small mammal assemblage after the exclusion of invasive herbivores and predators, similar to that found after vegetation clearance from fire [19], mining [48], agriculture [20] and deglaciation [22]. Over the 26 years, species diversity increased, five species responded by increasing in abundance, and two rodent species broadened their distribution into non-preferred habitats. Animal succession is thought to relate to successional changes in vegetation density [49], cover [50], and patchiness of vegetation structure and composition [20], with Fox’s habitat accommodation model (1990) proposing that small mammals appear in a landscape once physical thresholds of suitable habitat are reached [51]. Despite some browsing damage by reintroduced herbivores [52], our study site did not undergo major changes in perennial vegetation cover [31] and we suggest that small mammal succession can also be triggered by removal of disturbance in the form of invasive predators. In this situation, succession occurred due to interspecific differences in the timing of reinvasion and then subsequent competitive interactions. Our results demonstrate that succession dynamics can be triggered by processes that suppress, alter or remove the existing small mammal community and are not just driven by changes in vegetation structure.

Interspecific differences in post-predator-exclusion population trajectories of rodents are likely linked to competition and differences in predator susceptibility affecting reinvasion time. Smaller rodent species responded earliest to the removal of invasive species, with significant increases within 2 years in the Bolam’s mouse and house mouse. This early response is influenced by their presence in the region at the time of reserve establishment, likely due to their smaller size allowing them to coexist with cats and foxes. The house mouse was the first small mammal to increase inside the reserve, consistent with its high fecundity and reputation as a colonizing species [53,54]. House mice were the first mammals to increase within a cat- and fox-free exclosure site in Western Australia within 2 years [14]. Both small rodent species responded within 12 months to large rainfall events prior to reserve establishment [34] suggesting they breed quickly and are able to take advantage of vacant niches. In terms of succession dynamics, these species acted like biological legacies [55], where their presence in the region at the time of reserve establishment affected their quick response. However, after an initial increase, house mice were only abundant in high rainfall years, lower inside the reserve, and there was evidence of competition from native species. Overall, these data support evidence that this species is a fugitive species that does not represent a significant competitive threat to most native rodents in Australia [47,56].

The larger rodent species, hopping mice and plains mice, were not known from within 50 km of the reserve at the start of the study [10,57] likely due to the susceptibility of mammal species over 35 g to cat and fox predation [23]. This was proven during their widespread recovery after Rabbit Haemorrhagic Disease Virus decimated rabbits, leading to a decline in cats and foxes [58]. These larger rodents, which are preferred prey of feral cats [59], became significantly more abundant inside the reserve at 5 (hopping mice) and 9 years (plains mice), suggesting their absence at the start of the study delayed their re-establishment. Succession deglaciation studies suggest that changes in wildlife communities are not just influenced by vegetation change but by dispersal capabilities [22,60]. In our study, differences in reinvasion timing were also due to lower regional abundance from increased vulnerability to introduced predators.

Once they reached the reserve and bred up after rain, hopping mice and plains mice dominated dune and swale sites, respectively, exerting significant competitive effects on smaller rodents and reducing their abundance. Large rodents exhibited rainfall-induced boom periods before becoming limited by intraspecific competition. Competition is a known mechanism driving successional shifts in community composition. Although succession-induced competition is usually linked to changes in vegetation favouring different species at different times, our increased competition was likely due to interspecific interference competition from larger rodents (as seen in [61]) and intraspecific exploitation competition due to limiting resources from high population density. Results highlight how interspecific and intraspecific competition is suppressed by invasive species. Other studies found competition can significantly change the relative abundance of small mammals in desert ecosystems [9]. Lima *et al*. [3] found the intensity of interspecific competition changed with rainfall, increasing during dry years in a Chilean desert. Previtali *et al*. [62] found two-thirds of the variation in the population rate of change in rodents in Chile was due to intraspecific competition.

Compared with rodents, dasyurids exhibited a muted response to the removal of invasive species supporting Dexter *et al*. [63] but contrasting with [12] who found dasyurids were fourfold higher inside a fenced reserve within 2 years. Although the stripe-faced dunnart eventually responded, this recovery was slow and most noticeable after 8 years. The similar-sized fat-tailed dunnart did not show significant responses to the exclusion treatment, despite being found to be an early successional species after mining [48] reportedly due to lack of competition and a tolerance to disturbance. As vegetation at our study site was not physically disturbed, stripe-faced dunnarts may have outcompeted fat-tailed dunnarts within the reserve, preventing them from being increasers in this context.

An interesting change inside the reserve was the expansion of some rodents into non-preferred habitats. Plains mice are commonly found in clay interdunal swales [41,47] but they expanded into adjacent dune areas, despite rarely being recorded on dunes outside. A similar trend was found for hopping mice that expanded into swale areas inside the reserve. This expansion of the realized niche of two rodent species is similar to other responses to lower predation levels found in rock wallabies [27], and supports Kotler [64] who found predation risk can significantly segregate habitat use in small mammal communities. Such niche expansion has conservation implications as threatened species conservation focuses on conserving realized rather than fundamental niches despite their importance for population expansion.

As expected, high rainfall triggered rapid growth in rodent abundance 18 months later. We found nonlinear relationships between predicted carrying capacity and rainfall, and suggest this is due to strong intraspecific rodent competition during high rainfall years inside, and the effects of predation outside. Other studies have found nonlinear relationships induced by environment–species interactions [38] and highlight the importance of accounting for both biotic and abiotic interactions when investigating community change [32]. Desert rodents are known boom-bust species due to their response to rainfall-driven pulses in seed and plant dietary material [2,4,65,66]. Conversely, we found limited effects of rainfall on small dasyurid abundance. Desert dasyurids are insectivorous and many smaller dasyurids are not as responsive to rainfall, possibly due to their opportunistic foraging strategy and flexible habitat requirements [67].

The relative influence of bottom-up and top-down effects on desert small mammals has long been discussed, with some suggesting resource availability has a greater influence than predation [1,62], and others suggesting shifting importance based on rainfall cycles [68]. Conversely, predator manipulation experiments have shown that predation limits vertebrate prey, and invasive predators have on average twice the impact of native predators [69]. Although populations both inside and outside the reserve responded to high rainfall events with increases in abundance, the exclusion of cats, foxes and rabbits led to peaks that were up to 33 times higher and persisted for longer. Most differences were 4−9 times higher inside, significantly higher than the 1.6-fold and 1.7-fold overall effect of predators on prey populations found during global meta-analyses [11,70]. Our results suggest the impact of invasive predators on native mammals may be as much as 10-fold higher than native predators, and much higher than triple the rate suggested by Salo *et al*. [69] for Australia.

The exclusion of rabbits can increase the cover of arid grasses and shrubs [71], which may benefit seed-eating rodents. Although some of the increase in small mammal abundance inside could be due to increased food resources, the absence of any significant changes in body mass, or breeding rates inside versus outside the reserve suggests that food resources are unlikely to have been considerably elevated. Instead, rainfall events contributed to higher productivity both inside and outside the reserve leading to changes in body mass in some species. Additionally, the immediate response of some rodent species occurred before resources are likely to have responded to any removal of herbivores. Finally, reintroduced native herbivores such as the burrowing bettong reached high abundance and caused browsing damage over the study period offsetting some of the benefits from introduced herbivore exclusion [52].

Our measure of breeding rates was based on the presence of subadults during trapping events. Most arid zone rodents are opportunistic breeders with some species at our site also showing seasonal breeding in spring and early summer [34]. We acknowledge that some breeding events would have been missed due to our annual sampling regime and that analysis of the effects of climatic-driven bottom-up effects on breeding rates is not comprehensive. However, the proportion of subadults in the population reached as high as 40% at times suggesting some breeding events were captured. While we attribute most of the changes in the small mammal community to exclusion of invasive predators and resource limitation by increasing rodents, increases in native predators such as goannas [72] and anecdotal increases in barn owls and snakes [73] could have disproportionately suppressed small mammal species inside the reserve. The western quoll (*Dasyurus geoffroyi*) was reintroduced to the reserve in 2018 and preys on mammals [74], potentially reducing small mammal abundance. However, quolls were excluded from the Main Exclosure which contained more than half the inside sites, and only 12 quolls were released meaning they were at low density during the study.

Our results reinforce that current small mammal assemblages in arid areas are likely very different to pre-European assemblages. Outside the fence, competitive interactions were rare due to population suppression by invasive predators. When invasive species were excluded, both interspecific and intraspecific competitive interactions became dominant. Pre-European assemblages would likely have included higher diversity and up to 30 times higher rodent abundance, with stronger competitive interactions. More than 60% of the local mammal fauna are locally or globally extinct in our study area including stick-nest rats, Gould’s mice (*P. gouldi*) and mulgaras (*Dasycercus cristicauda*). This likely explained why species diversity but not richness increased inside the fence, many species were simply not present to reinvade. Our study highlights the significant impact that invasive species have on arid Australia, not just on species extinctions but through suppressing competitive interactions and bottom-up processes. Invasive predator exclusion caused the removal of endogenous disturbance that triggered a small mammal succession response through differences in recolonization timing, interspecific competition based on body size, and intraspecific competition from increased abundance. Invasive predator exclusion increased the realized niche for small mammals, leading to expansion into other habitats. Large-scale, long-term exclusion experiments provide us with a unique insight into processes that structure small mammal communities.

## Data Availability

Data are available through OSF at [75]. The authors kindly ask for the opportunity to collaborate if datasets are used. Supplementary material is available online [76].
